# Tricarbonyl(chlorodiphenylstannyl){η^5^-[2-(dimethylamino)ethyl]cyclopenta­dienyl}molybdenum

**DOI:** 10.1107/S1600536809014329

**Published:** 2009-04-25

**Authors:** Paul J. Fischer, Kristina M. Krohn, Victor G. Young Jr

**Affiliations:** a Chemistry Department, Macalester College, 1600 Grand Avenue, Saint Paul, MN 55105, USA; b X-ray Crystallographic Laboratory, Department of Chemistry, University of Minnesota, 207 Pleasant Street SE, Minneapolis, MN 55455, USA

## Abstract

Reaction of the tricarbon­yl{η^5^-[2-(dimethyl­amino)eth­yl]cyclo­penta­dien­yl}molybdenum anion and dichlorido­diphenyl­stannane affords the title compound, [MoSn(C_6_H_5_)_2_Cl(C_9_H_14_N)(CO)_3_], which exhibits a four-legged piano-stool geometry with chlorido­diphenyl­stannyl ligands unperturbed by the pendant 2-(dimethyl­amino)ethyl groups. The Mo—Sn bond length [2.7584 (5) Å] and the distortion of the tetra­hedral tin coordination geometry are similar to those observed in related tin-substituted tricarbonyl­molybdenum and -tungsten complexes.

## Related literature

The synthesis of Mo(SnMe_2_Cl)(CO)_3_(η^5^-Cp) was reported by Patil & Graham (1966 ▶). This methodolgy was extended to prepare Mo(SnPh_2_Cl)(CO)_3_(η^5^-Cp) by Marks & Seyam (1974 ▶). Triphenyl­tin and tricyclo­hexyl­tin derivatives of [*M*(CO)_3_(η^5^-C_5_H_4_CH_2_CH_2_NMe_2_)]^−^ (*M* = Mo and W) were reported by Fischer *et al.* (2005 ▶). The structural characterization and reaction chemistry of a variety of half-sandwich molybdenum and tungsten chloro­stannyl complexes have been reported by Braunschweig *et al.* (2007 ▶, 2009 ▶). The Lewis acidity of coordinatively saturated chloro­stannyl ligands has been explored by Tang *et al.* (2005 ▶). Structural parameters that define four-legged piano-stool geometries were detailed by Kubácek *et al.* (1982 ▶).
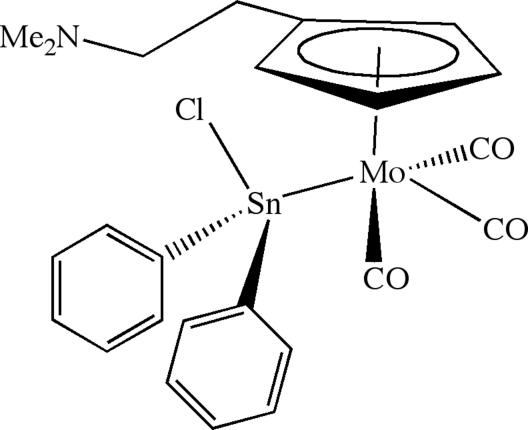


## Experimental

### 

#### Crystal data

[MoSn(C_6_H_5_)_2_Cl(C_9_H_14_N)(CO)_3_]
*M* *_r_* = 624.52
Triclinic, 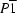

*a* = 8.8096 (8) Å
*b* = 9.3458 (8) Å
*c* = 16.1258 (14) Å
α = 89.392 (2)°
β = 85.269 (2)°
γ = 63.8560 (10)°
*V* = 1187.29 (18) Å^3^
*Z* = 2
Mo *K*α radiation
μ = 1.72 mm^−1^
*T* = 173 K
0.25 × 0.12 × 0.04 mm

#### Data collection

Bruker SMART CCD area-detector diffractometer
Absorption correction: multi-scan (*SADABS*; Bruker, 2003 ▶) *T*_min_ = 0.673, *T*_max_ = 0.935
14425 measured reflections
5431 independent reflections
4081 reflections with *I* > 2σ(*I*)
*R*_int_ = 0.056

#### Refinement

*R*[*F* ^2^ > 2σ(*F* ^2^)] = 0.036
*wR*(*F* ^2^) = 0.083
*S* = 1.00
5431 reflections
282 parameters
H-atom parameters constrained
Δρ_max_ = 0.71 e Å^−3^
Δρ_min_ = −0.56 e Å^−3^

### 

Data collection: *SMART* (Bruker, 2003 ▶); cell refinement: *SAINT* (Bruker, 2003 ▶); data reduction: *SAINT*; program(s) used to solve structure: *SHELXS97* (Sheldrick, 2008 ▶); program(s) used to refine structure: *SHELXL97* (Sheldrick, 2008 ▶); molecular graphics: *SHELXTL* (Sheldrick, 2008 ▶); software used to prepare material for publication: *SHELXTL*.

## Supplementary Material

Crystal structure: contains datablocks I, global. DOI: 10.1107/S1600536809014329/si2168sup1.cif

Structure factors: contains datablocks I. DOI: 10.1107/S1600536809014329/si2168Isup2.hkl

Additional supplementary materials:  crystallographic information; 3D view; checkCIF report

## Figures and Tables

**Table 1:** Selected bond lengths (Å)

Sn1—C13	2.144 (4)
Sn1—C19	2.146 (4)
Sn1—Cl1	2.3914 (11)

**Fig. 1.**
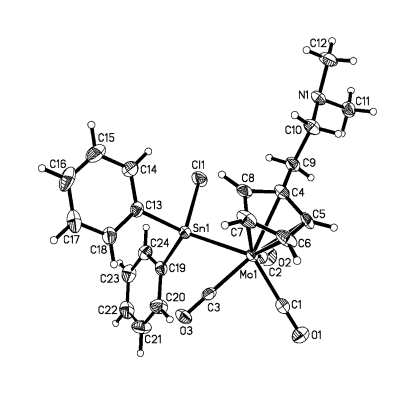
Molecular structure of 1 (50% thermal ellipsoids).

## supplementary crystallographic information

### Comment

The chemistry of half-sandwich molybdenum and tungsten chlorostannyl complexes is undergoing a revival since their discovery roughly forty years ago (Braunschweig *et al.*, 2009). Chlorostannyl ligands permit functionalization at tin in heterobimetallic complexes (Braunschweig *et al.*, 2007). The design of hypervalent tin ligands *via* intramolecular coordination of cyclopentadienyl-bound tethered donor groups is facilitated by chlorostannyl ligand Lewis acidity (Tang *et al.*, 2005).

The complexes W(SnBu_2_Cl)(CO)_3_(η^5^-Cp) (2) and W(SnMe_2_Cl)(CO)_3_(η^5^-Cp*) (3) were structurally characterized by Braunschweig *et al.* (2007). The title complex 1 is the first crystallographically characterized substance of general formula *M*(SnAr_2_*X*)(CO)_3_(η^5^-Cp).

The Mo—Sn bond length in 1 is slightly shorter (Table 1) than the W—Sn distances found in 2 (2.7959 (5) Å) and 3 (2.7866 (3) Å), respectively. The steric impact of the SnPh_3_ ligand relative to SnPh_2_Cl is minimal on the basis of Mo—Sn bond lengths; this separation is 2.8152 (3) Å in Mo(SnPh_3_)(CO)_3_(η^5^-C_5_H_4_CH_2_CH_2_NMe_2_) (4) (Fischer *et al.*, 2005). The geometric parameters of 1-4 satisfy the requirements for four-legged piano-stool structures (Kubácek *et al.*, 1982).

The tetrahedral coordination geometry of the tin atoms in 1-3 are distorted more significantly than that of 4. In 4, the diagnostic six angles about tin range from 105.60 (9)° to 116.15 (6)°, while the corresponding angles in 1 range from 100.30 (11)° to 120.34 (11)°. In 1-3 the M—Sn—C angles are widened whereas the C—Sn—Cl angles are more acute in comparison to the ideal tetrahedral angle. The angles about the Sn atoms of 1 and 3 are very similar as the phenyl and methyl substituents, respectively, attempt to minimize steric hinderance with the *M*(CO)_3_(Cp) fragment. The C(13)—Sn(1)—Mo (116.32 (10)°) and C(19)—Sn(1)—Mo (120.34 (11)°) angles in 1 are similar to the corresponding angles found in 3 (118.56 (11), 118.31 (12)°). The widening of these angles is accommodated by compression of C—Sn—Cl angles. These angles in 1 (C(13)—Sn(1)—Cl(1), 100.30 (11)°; C(19)—Sn(1)—Cl(1), 101.25 (11)°) are similar to the corresponding angles found in 3 (99.75 (12), 100.48 (13)°).

### Experimental

The following procedures were conducted under argon using standard techniques for handling air and moisture sensitive substances. THF (75 ml) was added to molybdenum hexacarbonyl (0.415 g, 1.57 mmol) and sodium ((2-dimethylamino)ethyl)cyclopentadienide (0.300 g, 1.88 mmol). The yellow solution was refluxed for 15 h. Addition of dichlorodiphenylstannane (0.540 g, 1.57 mmol) in THF (30 ml) resulted in a pale yellow suspension. A single molybdenum carbonyl complex was present in solution (based on IR spectroscopy) within 10 min. The suspension was filtered through Celite. THF was removed *in vacuo* revealing a yellow oil. The product was extracted with pentane (5 * 70 ml). Each colorless extract was filtered. The combined extracts were concentrated *in vacuo* until a yellow solid precipitated. The suspension (75 ml) was cooled and filtered at -70°C. The pale yellow, moderately air-sensitive solid was washed with pentane (-60°C, 5 * 15 ml) and dried *in vacuo*. Recrystallization from pentane at -65°C afforded pale yellow microcrystals (0.461 g, 47%). X-ray quality single crystals of 1 were obtained from a supersaturated pentane solution.

### Refinement

The structure was solved using Bruker *SHELXTL* and refined using Bruker *SHELXTL*. The space group was determined based on systematic absences and intensity statistics. A direct-methods solution was calculated which provided most non-hydrogen atoms from the E-map. Full-matrix least squares / difference Fourier cycles were performed which located the remaining non-hydrogen atoms. All non-hydrogen atoms were refined with anisotropic displacement parameters. All hydrogen atoms were placed in ideal positions and refined as riding atoms with relative isotropic displacement parameters. The final full matrix least squares refinement converged to *R*1 = 0.0362 and *wR*2 = 0.0827 (F^2^, all data).

### Crystal data

**Table:**

[MoSn(C_6_H_5_)_2_Cl(C_9_H_14_N)(CO)_3_]	*Z* = 2
*M**_r_* = 624.52	*F*(000) = 616
Triclinic, *P*1	*D*_x_ = 1.747 Mg m^−^^3^
Hall symbol: -P 1	Mo *K*α radiation, λ = 0.71073 Å
*a* = 8.8096 (8) Å	Cell parameters from 2659 reflections
*b* = 9.3458 (8) Å	θ = 2.4–27.4°
*c* = 16.1258 (14) Å	µ = 1.72 mm^−^^1^
α = 89.392 (2)°	*T* = 173 K
β = 85.269 (2)°	Plate, colourless
γ = 63.856 (1)°	0.25 × 0.12 × 0.04 mm
*V* = 1187.29 (18) Å^3^	

### Data collection

**Table:**

Bruker SMART CCD area-detector diffractometer	5431 independent reflections
Radiation source: sealed tube	4081 reflections with *I* > 2σ(*I*)
graphite	*R*_int_ = 0.056
φ and ω scans	θ_max_ = 27.5°, θ_min_ = 2.4°
Absorption correction: multi-scan (*SADABS*; Bruker, 2003)	*h* = −11→11
*T*_min_ = 0.673, *T*_max_ = 0.935	*k* = −12→12
14425 measured reflections	*l* = 0→20

### Refinement

**Table:**

Refinement on *F*^2^	Primary atom site location: structure-invariant direct methods
Least-squares matrix: full	Secondary atom site location: difference Fourier map
*R*[*F*^2^ > 2σ(*F*^2^)] = 0.036	Hydrogen site location: inferred from neighbouring sites
*wR*(*F*^2^) = 0.083	H-atom parameters constrained
*S* = 1.00	*w* = 1/[σ^2^(*F*_o_^2^) + (0.0315*P*)^2^] where *P* = (*F*_o_^2^ + 2*F*_c_^2^)/3
5431 reflections	(Δ/σ)_max_ = 0.001
282 parameters	Δρ_max_ = 0.71 e Å^−^^3^
0 restraints	Δρ_min_ = −0.56 e Å^−^^3^

### Special details

**Table:**

Experimental. Anal. Calcd for C_24_H_24_ClMoNO_3_Sn: C, 46.15; H, 3.87; N, 2.24. Found: C, 46.01, H, 3.78; N, 2.30.IR (THF) ν(CO): 2011 (*s*), 1944 (m, sh), 1912 (*s*) cm^-1^; (pentane) ν(CO): 2015 (*s*), 1952 (*m*), 1919 (*m*) cm^-1^; (nujol) ν(CO): 2011 (*s*), 1942 (m, sh), 1893 (*s*) cm^-1^.NMR (CD_2_Cl_2_, 300 MHz) ^1^H δ 7.78–7.54 (m, 4H, *ortho*, SnPh_2_Cl), 7.48–7.32 (m, 6H, *meta*/*para*, SnPh_2_Cl), 5.52 (app. t, J=2.1 Hz, 2H, Cp), 5.27 (app. t, J=2.1 Hz, 2H, Cp), 2.44–2.29 (m, 4H, CH_2_CH_2_), 2.14 (s, 6H, CH_3_).NMR (CD_2_Cl_2_, 75.5 MHz) ^13^C{^1^H} δ 228.64 (s, *trans* CO), 224.35 (s, *cis* CO), 146.11 (s, *ipso* C), 135.30 (s, *ortho* C, ^117,119^Sn-^13^C sat. (merged): 135.63, 134.97, ^2^J_SnC_=49.7 Hz), 129.80 (s, *para*, ^117,119^Sn-^13^C sat. (merged): 129.89, 129.73, ^4^J_SnC_=12.2 Hz), 129.12 (s, *meta* C, ^117,119^Sn-^13^C sat. (merged): 129.48, 128.76, ^3^J_SnC_=54.5 Hz), 114.60 (s, quat. C, Cp), 91.75 (s, Cp), 89.65 (s, Cp), 61.15 (s, CH_2_*C*H_2_N(CH_3_)_2_), 45.57 (s, CH_2_CH_2_N(*C*H_3_)_2_), 27.08 (s, *C*H_2_CH_2_N(CH_3_)_2_).
Geometry. All e.s.d.'s (except the e.s.d. in the dihedral angle between two l.s. planes) are estimated using the full covariance matrix. The cell e.s.d.'s are taken into account individually in the estimation of e.s.d.'s in distances, angles and torsion angles; correlations between e.s.d.'s in cell parameters are only used when they are defined by crystal symmetry. An approximate (isotropic) treatment of cell e.s.d.'s is used for estimating e.s.d.'s involving l.s. planes.
Refinement. A crystal (approximate dimensions 1/4 *x* 0.12 *x* 0.04 mm^3^) was placed onto the tip of a 0.1 mm diameter glass capillary and mounted on a CCD area detector diffractometer for a data collection at 173 (2) K (*SMART*, Bruker, 2003). A preliminary set of cell constants was calculated from reflections harvested from three sets of 20 frames. These initial sets of frames were oriented such that orthogonal wedges of reciprocal space were surveyed. This produced initial orientation matrices determined from 48 reflections. The data collection was carried out using Mo *K*α radiation (graphite monochromator) with a frame time of 8 s and a detector distance of 4.8 cm. A randomly oriented region of reciprocal space was surveyed to the extent of one sphere and to a resolution of 0.77 Å. Four major sections of frames were collected with 0.30° steps in ω at four different φ settings and a detector position of -28° in 2θ. The intensity data were corrected for absorption and decay (*SADABS*, Bruker, 2003). Final cell constants were calculated from 2659 strong reflections from the actual data collection after integration (*SAINT*, Bruker, 2003).Refinement of *F*^2^ against ALL reflections. The weighted *R*-factor *wR* and goodness of fit *S* are based on *F*^2^, conventional *R*-factors *R* are based on *F*, with *F* set to zero for negative *F*^2^. The threshold expression of *F*^2^ > σ(*F*^2^) is used only for calculating *R*-factors(gt) *etc*. and is not relevant to the choice of reflections for refinement. *R*-factors based on *F*^2^ are statistically about twice as large as those based on *F*, and *R*- factors based on ALL data will be even larger.

### Fractional atomic coordinates and isotropic or equivalent isotropic displacement parameters (Å^2^)

**Table:**

	*x*	*y*	*z*	*U*_iso_*/*U*_eq_	
Mo1	0.58741 (4)	0.72843 (4)	0.66247 (2)	0.01938 (9)	
C1	0.6228 (5)	0.6492 (5)	0.5449 (3)	0.0282 (9)	
O1	0.6433 (4)	0.6021 (4)	0.47726 (19)	0.0434 (8)	
C2	0.8374 (5)	0.6518 (5)	0.6404 (3)	0.0265 (9)	
O2	0.9820 (4)	0.5983 (4)	0.6286 (2)	0.0420 (8)	
C3	0.4662 (5)	0.9349 (5)	0.6085 (2)	0.0223 (9)	
O3	0.3862 (3)	1.0542 (3)	0.57849 (17)	0.0279 (7)	
C4	0.6083 (5)	0.5692 (5)	0.7791 (2)	0.0242 (9)	
C5	0.5505 (5)	0.5124 (5)	0.7139 (3)	0.0267 (9)	
H5A	0.6093	0.4105	0.6868	0.032*	
C6	0.3894 (6)	0.6330 (5)	0.6953 (3)	0.0353 (11)	
H6A	0.3222	0.6263	0.6538	0.042*	
C7	0.3483 (5)	0.7632 (5)	0.7495 (3)	0.0338 (11)	
H7A	0.2470	0.8602	0.7514	0.041*	
C8	0.4815 (5)	0.7269 (5)	0.8010 (2)	0.0267 (9)	
H8A	0.4862	0.7953	0.8428	0.032*	
C9	0.7613 (5)	0.4754 (5)	0.8262 (3)	0.0273 (9)	
H9A	0.8019	0.5484	0.8498	0.033*	
H9B	0.8542	0.3974	0.7881	0.033*	
C10	0.7123 (5)	0.3881 (5)	0.8963 (3)	0.0296 (10)	
H10A	0.6087	0.4656	0.9289	0.035*	
H10B	0.6844	0.3072	0.8714	0.035*	
N1	0.8441 (4)	0.3094 (4)	0.9527 (2)	0.0262 (8)	
C11	0.9863 (5)	0.1694 (5)	0.9137 (3)	0.0304 (10)	
H11A	1.0350	0.2008	0.8641	0.046*	
H11B	0.9466	0.0916	0.8977	0.046*	
H11C	1.0730	0.1215	0.9531	0.046*	
C12	0.7735 (6)	0.2630 (5)	1.0275 (3)	0.0370 (11)	
H12A	0.6813	0.3579	1.0552	0.055*	
H12B	0.8626	0.2116	1.0654	0.055*	
H12C	0.7293	0.1883	1.0121	0.055*	
Sn1	0.66959 (3)	0.95053 (3)	0.732290 (16)	0.02009 (8)	
Cl1	0.86682 (14)	0.82366 (13)	0.83383 (7)	0.0336 (3)	
C13	0.4703 (5)	1.1368 (5)	0.8078 (2)	0.0235 (9)	
C14	0.4566 (6)	1.1328 (5)	0.8939 (3)	0.0321 (10)	
H14A	0.5345	1.0426	0.9209	0.038*	
C15	0.3298 (6)	1.2593 (6)	0.9416 (3)	0.0418 (12)	
H15A	0.3210	1.2541	1.0005	0.050*	
C16	0.2193 (6)	1.3896 (6)	0.9037 (3)	0.0450 (13)	
H16A	0.1352	1.4767	0.9364	0.054*	
C17	0.2282 (6)	1.3965 (5)	0.8176 (3)	0.0396 (12)	
H17A	0.1492	1.4872	0.7914	0.047*	
C18	0.3534 (5)	1.2702 (5)	0.7697 (3)	0.0301 (10)	
H18A	0.3592	1.2747	0.7107	0.036*	
C19	0.7998 (5)	1.0601 (4)	0.6573 (2)	0.0234 (9)	
C20	0.7633 (6)	1.1042 (5)	0.5768 (3)	0.0353 (11)	
H20A	0.6844	1.0783	0.5517	0.042*	
C21	0.8401 (6)	1.1859 (6)	0.5316 (3)	0.0410 (12)	
H21A	0.8138	1.2145	0.4760	0.049*	
C22	0.9535 (6)	1.2252 (5)	0.5667 (3)	0.0379 (12)	
H22A	1.0046	1.2824	0.5360	0.045*	
C23	0.9928 (6)	1.1815 (5)	0.6468 (3)	0.0347 (11)	
H23A	1.0730	1.2068	0.6711	0.042*	
C24	0.9161 (5)	1.1009 (5)	0.6922 (3)	0.0286 (10)	
H24A	0.9428	1.0728	0.7477	0.034*	

### Atomic displacement parameters (Å^2^)

**Table:**

	*U*^11^	*U*^22^	*U*^33^	*U*^12^	*U*^13^	*U*^23^
Mo1	0.02176 (19)	0.01690 (18)	0.01947 (19)	−0.00855 (15)	−0.00209 (14)	0.00347 (14)
C1	0.035 (3)	0.022 (2)	0.028 (2)	−0.012 (2)	−0.0045 (19)	0.0042 (18)
O1	0.061 (2)	0.0359 (19)	0.0279 (18)	−0.0161 (17)	−0.0040 (16)	−0.0046 (15)
C2	0.030 (2)	0.022 (2)	0.028 (2)	−0.0111 (19)	−0.0049 (19)	0.0037 (17)
O2	0.0278 (18)	0.045 (2)	0.048 (2)	−0.0117 (16)	0.0004 (15)	−0.0022 (16)
C3	0.027 (2)	0.026 (2)	0.016 (2)	−0.0140 (19)	−0.0008 (17)	−0.0027 (17)
O3	0.0258 (16)	0.0235 (15)	0.0288 (16)	−0.0053 (13)	−0.0058 (13)	0.0093 (13)
C4	0.023 (2)	0.024 (2)	0.027 (2)	−0.0111 (18)	−0.0017 (17)	0.0083 (17)
C5	0.032 (2)	0.019 (2)	0.035 (2)	−0.0164 (19)	−0.0063 (19)	0.0107 (18)
C6	0.031 (2)	0.042 (3)	0.042 (3)	−0.023 (2)	−0.013 (2)	0.023 (2)
C7	0.021 (2)	0.033 (3)	0.039 (3)	−0.005 (2)	0.0062 (19)	0.014 (2)
C8	0.033 (2)	0.025 (2)	0.020 (2)	−0.0130 (19)	0.0052 (18)	0.0064 (17)
C9	0.032 (2)	0.026 (2)	0.029 (2)	−0.018 (2)	−0.0093 (19)	0.0076 (18)
C10	0.026 (2)	0.029 (2)	0.034 (2)	−0.0116 (19)	−0.0073 (19)	0.0117 (19)
N1	0.0263 (19)	0.0221 (18)	0.0282 (19)	−0.0085 (15)	−0.0057 (15)	0.0090 (15)
C11	0.034 (3)	0.022 (2)	0.034 (3)	−0.010 (2)	−0.009 (2)	0.0043 (18)
C12	0.042 (3)	0.036 (3)	0.029 (2)	−0.015 (2)	−0.002 (2)	0.014 (2)
Sn1	0.02179 (15)	0.01760 (14)	0.02049 (15)	−0.00814 (12)	−0.00317 (11)	0.00216 (11)
Cl1	0.0381 (6)	0.0298 (6)	0.0355 (6)	−0.0152 (5)	−0.0179 (5)	0.0096 (5)
C13	0.024 (2)	0.022 (2)	0.027 (2)	−0.0132 (18)	0.0016 (17)	−0.0024 (17)
C14	0.037 (3)	0.032 (2)	0.027 (2)	−0.016 (2)	−0.001 (2)	−0.0019 (19)
C15	0.047 (3)	0.051 (3)	0.030 (3)	−0.026 (3)	0.011 (2)	−0.012 (2)
C16	0.038 (3)	0.038 (3)	0.055 (3)	−0.018 (2)	0.023 (2)	−0.024 (3)
C17	0.034 (3)	0.022 (2)	0.058 (3)	−0.009 (2)	0.003 (2)	−0.002 (2)
C18	0.032 (2)	0.023 (2)	0.034 (2)	−0.013 (2)	0.0053 (19)	−0.0018 (19)
C19	0.021 (2)	0.019 (2)	0.027 (2)	−0.0069 (17)	0.0012 (17)	0.0003 (17)
C20	0.037 (3)	0.042 (3)	0.032 (3)	−0.021 (2)	−0.010 (2)	0.008 (2)
C21	0.050 (3)	0.053 (3)	0.026 (2)	−0.028 (3)	−0.003 (2)	0.011 (2)
C22	0.042 (3)	0.030 (2)	0.041 (3)	−0.019 (2)	0.016 (2)	0.001 (2)
C23	0.030 (2)	0.034 (3)	0.044 (3)	−0.020 (2)	0.006 (2)	−0.006 (2)
C24	0.025 (2)	0.029 (2)	0.030 (2)	−0.0112 (19)	−0.0011 (18)	−0.0022 (19)

### Geometric parameters (Å, °)

**Table:**

Mo1—C3	1.981 (4)	C11—H11B	0.9800
Mo1—C1	1.992 (4)	C11—H11C	0.9800
Mo1—C2	1.994 (4)	C12—H12A	0.9800
Mo1—C6	2.308 (4)	C12—H12B	0.9800
Mo1—C5	2.317 (4)	C12—H12C	0.9800
Mo1—C7	2.331 (4)	Sn1—C13	2.144 (4)
Mo1—C8	2.351 (4)	Sn1—C19	2.146 (4)
Mo1—C4	2.353 (4)	Sn1—Cl1	2.3914 (11)
Mo1—Sn1	2.7584 (5)	C13—C14	1.384 (6)
C1—O1	1.150 (5)	C13—C18	1.396 (6)
C2—O2	1.145 (5)	C14—C15	1.397 (6)
C3—O3	1.152 (4)	C14—H14A	0.9500
C4—C5	1.408 (6)	C15—C16	1.357 (7)
C4—C8	1.428 (5)	C15—H15A	0.9500
C4—C9	1.509 (5)	C16—C17	1.386 (7)
C5—C6	1.424 (6)	C16—H16A	0.9500
C5—H5A	0.9500	C17—C18	1.392 (6)
C6—C7	1.399 (6)	C17—H17A	0.9500
C6—H6A	0.9500	C18—H18A	0.9500
C7—C8	1.409 (6)	C19—C20	1.379 (6)
C7—H7A	0.9500	C19—C24	1.398 (5)
C8—H8A	0.9500	C20—C21	1.390 (6)
C9—C10	1.530 (5)	C20—H20A	0.9500
C9—H9A	0.9900	C21—C22	1.369 (6)
C9—H9B	0.9900	C21—H21A	0.9500
C10—N1	1.457 (5)	C22—C23	1.376 (6)
C10—H10A	0.9900	C22—H22A	0.9500
C10—H10B	0.9900	C23—C24	1.384 (6)
N1—C11	1.456 (5)	C23—H23A	0.9500
N1—C12	1.461 (5)	C24—H24A	0.9500
C11—H11A	0.9800		
			
C3—Mo1—C1	81.02 (16)	C4—C9—C10	109.1 (3)
C3—Mo1—C2	109.86 (16)	C4—C9—H9A	109.9
C1—Mo1—C2	79.28 (17)	C10—C9—H9A	109.9
C3—Mo1—C6	106.33 (16)	C4—C9—H9B	109.9
C1—Mo1—C6	91.77 (17)	C10—C9—H9B	109.9
C2—Mo1—C6	140.66 (16)	H9A—C9—H9B	108.3
C3—Mo1—C5	141.90 (15)	N1—C10—C9	114.0 (3)
C1—Mo1—C5	93.04 (16)	N1—C10—H10A	108.8
C2—Mo1—C5	105.82 (15)	C9—C10—H10A	108.8
C6—Mo1—C5	35.87 (14)	N1—C10—H10B	108.8
C3—Mo1—C7	92.95 (15)	C9—C10—H10B	108.8
C1—Mo1—C7	122.26 (17)	H10A—C10—H10B	107.6
C2—Mo1—C7	151.56 (16)	C11—N1—C10	112.1 (3)
C6—Mo1—C7	35.08 (16)	C11—N1—C12	109.4 (3)
C5—Mo1—C7	58.61 (15)	C10—N1—C12	110.0 (3)
C3—Mo1—C8	113.36 (15)	N1—C11—H11A	109.5
C1—Mo1—C8	149.29 (16)	N1—C11—H11B	109.5
C2—Mo1—C8	117.21 (16)	H11A—C11—H11B	109.5
C6—Mo1—C8	58.71 (16)	N1—C11—H11C	109.5
C5—Mo1—C8	58.52 (15)	H11A—C11—H11C	109.5
C7—Mo1—C8	35.02 (15)	H11B—C11—H11C	109.5
C3—Mo1—C4	148.50 (15)	N1—C12—H12A	109.5
C1—Mo1—C4	124.20 (15)	N1—C12—H12B	109.5
C2—Mo1—C4	94.55 (15)	H12A—C12—H12B	109.5
C6—Mo1—C4	59.16 (14)	N1—C12—H12C	109.5
C5—Mo1—C4	35.09 (14)	H12A—C12—H12C	109.5
C7—Mo1—C4	58.64 (14)	H12B—C12—H12C	109.5
C8—Mo1—C4	35.36 (13)	C13—Sn1—C19	106.86 (14)
C3—Mo1—Sn1	71.39 (11)	C13—Sn1—Cl1	100.30 (11)
C1—Mo1—Sn1	129.99 (12)	C19—Sn1—Cl1	101.25 (11)
C2—Mo1—Sn1	72.45 (11)	C13—Sn1—Mo1	116.32 (10)
C6—Mo1—Sn1	135.31 (13)	C19—Sn1—Mo1	120.34 (11)
C5—Mo1—Sn1	133.76 (11)	Cl1—Sn1—Mo1	108.86 (3)
C7—Mo1—Sn1	100.50 (12)	C14—C13—C18	118.4 (4)
C8—Mo1—Sn1	80.70 (10)	C14—C13—Sn1	122.3 (3)
C4—Mo1—Sn1	98.79 (10)	C18—C13—Sn1	119.3 (3)
O1—C1—Mo1	179.4 (4)	C13—C14—C15	121.0 (4)
O2—C2—Mo1	175.7 (4)	C13—C14—H14A	119.5
O3—C3—Mo1	175.6 (3)	C15—C14—H14A	119.5
C5—C4—C8	107.1 (4)	C16—C15—C14	120.0 (4)
C5—C4—C9	127.1 (4)	C16—C15—H15A	120.0
C8—C4—C9	125.3 (4)	C14—C15—H15A	120.0
C5—C4—Mo1	71.0 (2)	C15—C16—C17	120.5 (4)
C8—C4—Mo1	72.2 (2)	C15—C16—H16A	119.8
C9—C4—Mo1	128.7 (3)	C17—C16—H16A	119.8
C4—C5—C6	108.7 (4)	C16—C17—C18	119.8 (4)
C4—C5—Mo1	73.9 (2)	C16—C17—H17A	120.1
C6—C5—Mo1	71.7 (2)	C18—C17—H17A	120.1
C4—C5—H5A	125.7	C17—C18—C13	120.4 (4)
C6—C5—H5A	125.7	C17—C18—H18A	119.8
Mo1—C5—H5A	120.5	C13—C18—H18A	119.8
C7—C6—C5	107.4 (4)	C20—C19—C24	117.8 (4)
C7—C6—Mo1	73.4 (2)	C20—C19—Sn1	122.2 (3)
C5—C6—Mo1	72.4 (2)	C24—C19—Sn1	119.8 (3)
C7—C6—H6A	126.3	C19—C20—C21	121.1 (4)
C5—C6—H6A	126.3	C19—C20—H20A	119.4
Mo1—C6—H6A	119.8	C21—C20—H20A	119.4
C6—C7—C8	108.9 (4)	C22—C21—C20	120.4 (4)
C6—C7—Mo1	71.6 (2)	C22—C21—H21A	119.8
C8—C7—Mo1	73.2 (2)	C20—C21—H21A	119.8
C6—C7—H7A	125.5	C21—C22—C23	119.6 (4)
C8—C7—H7A	125.5	C21—C22—H22A	120.2
Mo1—C7—H7A	121.3	C23—C22—H22A	120.2
C7—C8—C4	107.9 (4)	C22—C23—C24	120.3 (4)
C7—C8—Mo1	71.7 (2)	C22—C23—H23A	119.9
C4—C8—Mo1	72.4 (2)	C24—C23—H23A	119.9
C7—C8—H8A	126.0	C23—C24—C19	120.9 (4)
C4—C8—H8A	126.0	C23—C24—H24A	119.6
Mo1—C8—H8A	121.5	C19—C24—H24A	119.6
			
C3—Mo1—C1—O1	−152 (36)	C4—Mo1—C7—C6	−79.8 (3)
C2—Mo1—C1—O1	96 (36)	Sn1—Mo1—C7—C6	−173.8 (2)
C6—Mo1—C1—O1	−46 (36)	C3—Mo1—C7—C8	−128.2 (3)
C5—Mo1—C1—O1	−10 (36)	C1—Mo1—C7—C8	150.6 (2)
C7—Mo1—C1—O1	−64 (36)	C2—Mo1—C7—C8	15.9 (5)
C8—Mo1—C1—O1	−31 (36)	C6—Mo1—C7—C8	117.2 (4)
C4—Mo1—C1—O1	7(36)	C5—Mo1—C7—C8	78.7 (3)
Sn1—Mo1—C1—O1	152 (36)	C4—Mo1—C7—C8	37.4 (2)
C3—Mo1—C2—O2	−157 (5)	Sn1—Mo1—C7—C8	−56.6 (2)
C1—Mo1—C2—O2	−81 (5)	C6—C7—C8—C4	−0.8 (4)
C6—Mo1—C2—O2	−1(5)	Mo1—C7—C8—C4	−63.9 (3)
C5—Mo1—C2—O2	9(5)	C6—C7—C8—Mo1	63.1 (3)
C7—Mo1—C2—O2	61 (5)	C5—C4—C8—C7	0.5 (4)
C8—Mo1—C2—O2	72 (5)	C9—C4—C8—C7	−171.4 (4)
C4—Mo1—C2—O2	43 (5)	Mo1—C4—C8—C7	63.4 (3)
Sn1—Mo1—C2—O2	141 (5)	C5—C4—C8—Mo1	−62.9 (3)
C1—Mo1—C3—O3	98 (4)	C9—C4—C8—Mo1	125.2 (4)
C2—Mo1—C3—O3	173 (4)	C3—Mo1—C8—C7	58.8 (3)
C6—Mo1—C3—O3	9(4)	C1—Mo1—C8—C7	−54.5 (4)
C5—Mo1—C3—O3	14 (4)	C2—Mo1—C8—C7	−171.6 (3)
C7—Mo1—C3—O3	−24 (4)	C6—Mo1—C8—C7	−36.7 (2)
C8—Mo1—C3—O3	−54 (4)	C5—Mo1—C8—C7	−79.0 (3)
C4—Mo1—C3—O3	−48 (4)	C4—Mo1—C8—C7	−116.4 (4)
Sn1—Mo1—C3—O3	−125 (4)	Sn1—Mo1—C8—C7	123.7 (2)
C3—Mo1—C4—C5	107.3 (3)	C3—Mo1—C8—C4	175.1 (2)
C1—Mo1—C4—C5	−31.1 (3)	C1—Mo1—C8—C4	61.9 (4)
C2—Mo1—C4—C5	−111.3 (3)	C2—Mo1—C8—C4	−55.2 (3)
C6—Mo1—C4—C5	37.6 (3)	C6—Mo1—C8—C4	79.6 (3)
C7—Mo1—C4—C5	78.8 (3)	C5—Mo1—C8—C4	37.3 (2)
C8—Mo1—C4—C5	115.9 (3)	C7—Mo1—C8—C4	116.4 (4)
Sn1—Mo1—C4—C5	175.8 (2)	Sn1—Mo1—C8—C4	−119.9 (2)
C3—Mo1—C4—C8	−8.6 (4)	C5—C4—C9—C10	−84.0 (5)
C1—Mo1—C4—C8	−147.0 (3)	C8—C4—C9—C10	86.2 (5)
C2—Mo1—C4—C8	132.9 (3)	Mo1—C4—C9—C10	−178.8 (3)
C6—Mo1—C4—C8	−78.2 (3)	C4—C9—C10—N1	−172.7 (3)
C5—Mo1—C4—C8	−115.9 (3)	C9—C10—N1—C11	−71.6 (4)
C7—Mo1—C4—C8	−37.0 (2)	C9—C10—N1—C12	166.5 (4)
Sn1—Mo1—C4—C8	60.0 (2)	C3—Mo1—Sn1—C13	69.08 (17)
C3—Mo1—C4—C9	−129.9 (4)	C1—Mo1—Sn1—C13	129.3 (2)
C1—Mo1—C4—C9	91.7 (4)	C2—Mo1—Sn1—C13	−172.07 (17)
C2—Mo1—C4—C9	11.6 (4)	C6—Mo1—Sn1—C13	−25.59 (19)
C6—Mo1—C4—C9	160.4 (4)	C5—Mo1—Sn1—C13	−76.73 (19)
C5—Mo1—C4—C9	122.8 (5)	C7—Mo1—Sn1—C13	−20.51 (16)
C7—Mo1—C4—C9	−158.3 (4)	C8—Mo1—Sn1—C13	−49.54 (16)
C8—Mo1—C4—C9	−121.3 (5)	C4—Mo1—Sn1—C13	−80.05 (15)
Sn1—Mo1—C4—C9	−61.3 (4)	C3—Mo1—Sn1—C19	−62.57 (17)
C8—C4—C5—C6	−0.1 (4)	C1—Mo1—Sn1—C19	−2.39 (19)
C9—C4—C5—C6	171.6 (4)	C2—Mo1—Sn1—C19	56.28 (17)
Mo1—C4—C5—C6	−63.8 (3)	C6—Mo1—Sn1—C19	−157.24 (19)
C8—C4—C5—Mo1	63.7 (3)	C5—Mo1—Sn1—C19	151.62 (19)
C9—C4—C5—Mo1	−124.6 (4)	C7—Mo1—Sn1—C19	−152.15 (16)
C3—Mo1—C5—C4	−126.0 (3)	C8—Mo1—Sn1—C19	178.81 (16)
C1—Mo1—C5—C4	154.7 (3)	C4—Mo1—Sn1—C19	148.31 (16)
C2—Mo1—C5—C4	74.9 (3)	C3—Mo1—Sn1—Cl1	−178.60 (12)
C6—Mo1—C5—C4	−116.5 (4)	C1—Mo1—Sn1—Cl1	−118.42 (16)
C7—Mo1—C5—C4	−78.9 (3)	C2—Mo1—Sn1—Cl1	−59.75 (12)
C8—Mo1—C5—C4	−37.6 (2)	C6—Mo1—Sn1—Cl1	86.73 (15)
Sn1—Mo1—C5—C4	−5.7 (3)	C5—Mo1—Sn1—Cl1	35.59 (15)
C3—Mo1—C5—C6	−9.5 (4)	C7—Mo1—Sn1—Cl1	91.81 (11)
C1—Mo1—C5—C6	−88.8 (3)	C8—Mo1—Sn1—Cl1	62.78 (11)
C2—Mo1—C5—C6	−168.5 (3)	C4—Mo1—Sn1—Cl1	32.28 (10)
C7—Mo1—C5—C6	37.6 (3)	C19—Sn1—C13—C14	−123.0 (3)
C8—Mo1—C5—C6	78.9 (3)	Cl1—Sn1—C13—C14	−17.8 (3)
C4—Mo1—C5—C6	116.5 (4)	Mo1—Sn1—C13—C14	99.3 (3)
Sn1—Mo1—C5—C6	110.8 (3)	C19—Sn1—C13—C18	54.6 (3)
C4—C5—C6—C7	−0.4 (5)	Cl1—Sn1—C13—C18	159.8 (3)
Mo1—C5—C6—C7	−65.5 (3)	Mo1—Sn1—C13—C18	−83.1 (3)
C4—C5—C6—Mo1	65.1 (3)	C18—C13—C14—C15	−0.5 (6)
C3—Mo1—C6—C7	−71.1 (3)	Sn1—C13—C14—C15	177.1 (3)
C1—Mo1—C6—C7	−152.3 (3)	C13—C14—C15—C16	−0.9 (7)
C2—Mo1—C6—C7	132.6 (3)	C14—C15—C16—C17	1.7 (7)
C5—Mo1—C6—C7	115.0 (4)	C15—C16—C17—C18	−1.2 (7)
C8—Mo1—C6—C7	36.7 (2)	C16—C17—C18—C13	−0.2 (7)
C4—Mo1—C6—C7	78.2 (3)	C14—C13—C18—C17	1.1 (6)
Sn1—Mo1—C6—C7	8.7 (3)	Sn1—C13—C18—C17	−176.6 (3)
C3—Mo1—C6—C5	173.9 (3)	C13—Sn1—C19—C20	−96.5 (4)
C1—Mo1—C6—C5	92.7 (3)	Cl1—Sn1—C19—C20	158.9 (3)
C2—Mo1—C6—C5	17.6 (4)	Mo1—Sn1—C19—C20	39.0 (4)
C7—Mo1—C6—C5	−115.0 (4)	C13—Sn1—C19—C24	78.2 (3)
C8—Mo1—C6—C5	−78.3 (3)	Cl1—Sn1—C19—C24	−26.4 (3)
C4—Mo1—C6—C5	−36.8 (2)	Mo1—Sn1—C19—C24	−146.3 (3)
Sn1—Mo1—C6—C5	−106.3 (3)	C24—C19—C20—C21	0.3 (7)
C5—C6—C7—C8	0.7 (5)	Sn1—C19—C20—C21	175.1 (3)
Mo1—C6—C7—C8	−64.2 (3)	C19—C20—C21—C22	−0.4 (7)
C5—C6—C7—Mo1	64.9 (3)	C20—C21—C22—C23	0.9 (7)
C3—Mo1—C7—C6	114.6 (3)	C21—C22—C23—C24	−1.2 (7)
C1—Mo1—C7—C6	33.4 (3)	C22—C23—C24—C19	1.0 (6)
C2—Mo1—C7—C6	−101.3 (4)	C20—C19—C24—C23	−0.6 (6)
C5—Mo1—C7—C6	−38.5 (2)	Sn1—C19—C24—C23	−175.5 (3)
C8—Mo1—C7—C6	−117.2 (4)		
